# The relationship between ventricular dilatation, neuropathological and neurobehavioural changes in hydrocephalic rats

**DOI:** 10.1186/2045-8118-9-19

**Published:** 2012-09-01

**Authors:** Funmilayo Eniola Olopade, Matthew Temitayo Shokunbi, Anna-Leena Sirén

**Affiliations:** 1 Department of Anatomy, College of Medicine, University of Ibadan, Ibadan, Nigeria; 2 Department of Neurological Surgery, College of Medicine, University of Ibadan, Ibadan, Nigeria; 3 Department of Neurosurgery, University of Wuerzburg, 97080, Wuerzburg, Germany

**Keywords:** Hydrocephalus, Cognition, Neurobehavioural tests, Neuropathology, Cell death, Inflammation

## Abstract

**Background:**

The motor and cognitive deficits observed in hydrocephalus are thought to be due to axonal damage within the periventricular white matter. This study was carried out to investigate the relationship between ventricular size, cellular changes in brain, and neurobehavioural deficits in rats with experimental hydrocephalus.

**Methods:**

Hydrocephalus was induced in three-week old rats by intracisternal injection of kaolin. Behavioural and motor function were tested four weeks after hydrocephalus induction and correlated to ventricular enlargement which was classified into mild, moderate or severe. Gross brain morphology, routine histology and immunohistochemistry for oligodendrocytes (CNPase), microglia (Iba-1) and astrocytes (GFAP) were performed to assess the cellular changes.

**Results:**

Decreases in open field activity and forelimb grip strength in hydrocephalus correlated with the degree of ventriculomegaly. Learning in Morris water maze was significantly impaired in hydrocephalic rats. Gradual stretching of the ependymal layer, thinning of the corpus callosum, extracellular oedema and reduced cortical thickness were observed as the degree of ventriculomegaly increased. A gradual loss of oligodendrocytes in the corpus callosum and cerebral cortex was most marked in the severely-hydrocephalic brains, whereas the widespread astrogliosis especially in the subependymal layer was most marked in the brains with mild hydrocephalus. Retraction of microglial processes and increase in Iba-1 immunoreactivity in the white matter was associated ventriculomegaly.

**Conclusions:**

In hydrocephalic rats, oligodendrocyte loss, microglia activation, astrogliosis in cortical areas and thinning of the corpus callosum were associated with ventriculomegaly. The degree of ventriculomegaly correlated with motor and cognitive deficits.

## Background

Hydrocephalus is a relatively common neurological condition especially in children, occurring in 0.5 – 1 per 1,000 live births worldwide [1]. It is most usually characterized by an anomaly in the circulation of cerebrospinal fluid leading to its accumulation within the ventricles of the brain. The motor and cognitive deficits which occur in hydrocephalus are thought to be partly due to axonal damage within the periventricular white matter. In addition, myelin disruption is prominent in hydrocephalus [2], accounting for many of the neurological deficits in this disorder, thus necessitating an examination of the role of oligodendrocytes, the myelin-producing cells in the central nervous system.

The study of the full impact of these changes on behavior is necessary as the behavior of an organism represents the full functional integration of the nervous system [3]. It is reasonable to expect that the degree of ventricular dilatation in hydrocephalus will determine the span and severity of white matter injury and ultimately, functional deficits. However, previous studies of this relationship have revealed conflicting results. Lorber [4] concluded that even extreme ventricular dilation is compatible with normal physical and intellectual development into adult life after he examined a maths major student with an intelligent quotient (IQ) of 126 who had only a thin layer of cerebral cortex covering his ventricles. Similarly, Feuillet *et al*. [5] reported massive ventricular enlargement in a patient with normal social functioning and slightly reduced IQ of 75. He concluded, like Lorber, that the brain is able to adapt to the pathology due to a high level of redundancy in the normal brain function. In a subsequent study by Lorber, ventricular volumes (measured on CT scans) of hydrocephalic patients did not correlate well with their intelligence quotient [6]. Conversely, a study of the behavioural deficits in both chronic hydrocephalic humans and rats revealed an inverse relationship between ventricle volume and performance [7]. These conflicting results have prompted us to further examine the relationship between ventricular size and neurobehavioural deficits (locomotive, learning and memory defects), and to investigate the morphological changes observed in rats with experimental hydrocephalus.

## Methods

### Animals

A total of 87 three-week old rats were used for the study with 62 constituting the experimental group and 25 the control group. The animals were obtained from the animal holding facility of the department of Anatomy, University of Ibadan, Ibadan. All experiments were approved by the Ethical Review Board of the University of Ibadan. To induce hydrocephalus, the rats in the experimental group were anaesthetized with intraperitoneal injection of ketamine/xylazine (90/10 mg/kg), the skin of the dorsum of the neck was incised and the atlanto-occipital membrane was exposed. A sterile kaolin suspension, 0.02 ml (250 mg/ml in distilled water) was injected into the cistern magna with a 27-gauge needle. For the control rats, a sham procedure was performed in which the cisterna magna was punctured without fluid injection. The rats were housed in groups of 6 and given food and water *ad libitum*. The animals were weighed twice weekly and assessed for the development of hydrocephalus seen as increased head circumference, affected gait and dull general appearance. Sample photographs were taken of the control and hydrocephalic animals.

### Behavioural tests

A subset of 56 rats – 42 experimental and 14 controls underwent a series of behavioural tests (once for each rat) 4 weeks after the induction of hydrocephalus, to assess motor function, learning and memory.

#### Open field test

This test assesses general locomotive activity of rodents [8]. Each rat was placed in an open field, a 72 by 72 cm square box with lines on the floor dividing it into18 by 18 cm squares, for a period of 5 minutes and the following parameters were assessed: horizontal movements (measured by the number of transitions/lines crossed), vertical movement or rearing (the number of times the rat balances on only its hind feet), centre time (length of time spent in the centre square) and number of faecal boluses passed. All these parameters were assessed and manually recorded by the same set of observers.

#### Forelimb grip strength test

In this test, the forepaws were placed on a horizontally suspended metal wire 2 mm in diameter, 1 m in length and placed 1 m above a landing area filled with soft bedding. The length of time each rat was able to stay suspended before falling off the wire was recorded; a maximum of 2 minutes was given to each rat. This is a test of muscular strength in the forelimbs [9].

#### Morris water maze test

A modification of Morris water maze test was carried out to assess hippocampus-dependent spatial learning and memory [10]. This consisted of a circular pool of water 110 cm in diameter and 30 cm deep with a hidden circular escape platform (10 cm in diameter) which the rat must learn to locate using contextual and visual cues in the room. The pool was marked north, south, east and west and the hidden platform placed in a particular spot. Each rat was placed in the pool and expected to find the platform. If it did not find the platform after 60 seconds, the rat was guided to the platform and allowed to stay there for 15 seconds. Each rat went through this training twice. The length of time it took to find the platform was recorded. This test is a measure of learning ability. The test was repeated after a few hours and the rat’s ability to find the platform was recorded. This latter record is a test of its memory. The Morris water maze was introduced as an instrument with particular sensitivity to the effects of hippocampal lesions in rats [11]. It has been used previously to determine the extent to which hydrocephalic rats exhibit learning deficits and the effect of early ventricular shunting on the observed deficits [12].

### Measurement of ventricles

After undergoing behavioural tests, the rats were again anaesthesized with ketamine/xylazine (90/10 mg/kg) and perfused transcardially with 10% neutral buffered formalin. The brains were dissected out and post-fixed for 72 hours in the same solution. The brain was bisected in the coronal plane, at right angle to a horizontal tangent at the level of the optic chiasm. The surface of the distal half of the brain, so obtained was examined grossly and photographed using a Kodak M1063 digital camera. The thickness of the cortical mantle and the ventricular size were measured with digital vernier calipers. The ventricular diameter was measured as the maximum medio-lateral dimension of the frontal horn of the lateral ventricles. We classified hydrocephalus in this study into mild, moderate and severe, based on this measurement. Mild and moderate ventriculomegaly were defined as ventricular diameter less than and more than 1.5 mm respectively while severe ventriculomegaly was defined as visible separation / detachment of the cortical mantle from the caudate putamen with ventricular dilatation.

### Histology and immunostaining

A total of 23 brain samples were processed for paraffin embedding and sectioning: 6 controls, 5 mild, 6 moderate and 6 severe hydrocephalic brains. The brains were sectioned at 5 μm intervals. Selected sections were stained with hematoxylin and eosin (H&E) to demonstrate the general morphology of the brains at different stages/degrees of progression of hydrocephalus, and with luxol fast blue (LFB) counterstained with cresyl violet**,** to demonstrate white matter in the sections used for measurement of the thickness of the corpus callosum.

Immunohistological staining was performed for oligodendroctyes, astrocytes and microglial cells. The tissue sections were deparaffinized in xylene and rehydrated in decreasing concentrations of ethanol, then antigen retrieval was performed by boiling in citrate buffer (pH 6.0) for 30 min in the microwave oven. Non-specific antigens were blocked by preincubating for 1 h in 10% normal horse serum. The following primary antibodies were used: mouse monoclonal anti - 2', 3'-cyclic nucleotide 3'-phosphodiesterase (anti-CNPase, 1:2,000 dilution, Sigma-Aldrich, Hannover, Germany) for detecting oligodendrocytes, rabbit polyclonal anti-glial fibrillary acid protein (anti-GFAP, 1:1,000 dilution, Sigma-Aldrich) for detection of astrocytes and goat polyclonal ionized calcium binding adaptor molecule (Iba-1 C-20, 1:5,000 dilution, Wako Chemicals, Virginia, USA ) for detection of microglial cells. The tissue sections were incubated in primary antibodies overnight at 4°C except for the Iba-1 antibody which was incubated for 2 days. They were then incubated with appropriate biotin-conjugated secondary antibodies for 1 hour at room temperature followed by incubation with avidin-biotinylated horseradish peroxidase (Vectastain ABC kit, Vector Laboratories, California, USA) for 1 hour also at room temperature. The reaction product was revealed with 3-3’diaminobenzidine tetrahydrochloride (DAB) peroxidase substrate (Vector Laboratories, California, USA). The tissue sections were counterstained with hematoxylin stain, dehydrated, cleared and coverslipped with distyrene, plasticizer and xylene mixture (DPX).

### Analysis of sections

The sections were viewed with a Carl Zeiss light microscope (Carl Zeiss Microscopy GmbH, Göttingen, Germany). Cell counts were performed in the subependymal region and adjacent parietal cortex for the oligodendrocytes, astrocytes and microglia. The cells were counted manually from four random areas in each sample and the average count calculated. We counted neurons on the H&E- stained slides and measured the thickness of the corpus callosum on the luxol fast blue-stained slides using a calibrated eyepiece. Samples of the different groups –control, mild, moderate and severe hydrocephalus were selected for photomicrography. The photomicrographs were produced with a Carl Zeiss imaging microscope equipped with a Spot Insight digital camera (Carl Zeiss Microscopy GmbH, Göttingen, Germany). The images were captured unto a computer with Metavue computer software (Molecular devices LLC, California, USA).

### Data analysis

Data from the behavioural tests and the quantitative data from the tissue sections were statistically analyzed using the GraphPad Prism version 4.00 for Windows, GraphPad Software (San Diego, California, USA). Sample means generated after a statistical test to ascertain normal distribution, were compared among the various groups using analysis of variance (ANOVA) and Student t- test with confidence interval calculated at 95% and level of significance fixed at 5%. Pearson’s correlation test was also carried out with the software and level of significance fixed at 5%.

## Results

A total of 87 three-week old rats were used for the study, 62 experimental and 25 controls. Eighteen rats died during the study, eight of which were immediately post intracisternal injection due to possible brainstem damage and anaesthetic reaction. The others died within three days of injection due to spinal cord damage and subdural haemorrhage.

### Physical observations

**Figure 1:**
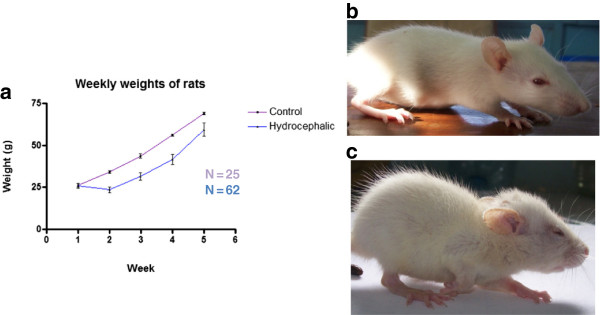
**Comparison of hydrocephalic rat and control. a:** Graph of body weights measured weekly in hydrocephalic and control rats. Hydrocephalus was induced in three-week old rats, designated week 1 on graph. After an initial loss of weight, the hydrocephalic rats did not regain the same weight as the controls ( *p<*0.05). Data are means ± SEM. **b:** control rat and **c:** hydrocephalic rat; note the dome-shaped head, hunched back and splayed limbs in the hydrocephalic rat.

The hydrocephalic rats lost body weight within the first week of induction of hydrocephalus. Although they gained over time, they were consistently lighter than the controls (F = 7.121, *p<*0.05, Figure 1a). Hydrocephalic rats also exhibited a general reduction in activity and food intake. The hydrocephalic rats developed an enlarged, dome-shaped head usually detectable within one week of kaolin injection. They also developed varying degrees of unsteady gait, lethargic pace and hunched back; impaired grooming resulted in a scruffy fur (Figure 1b and c). Six of them (about 10%) developed proptosis (protrusion of both eyeballs) and conjuctival hyperaemia.

**Figure 2:**
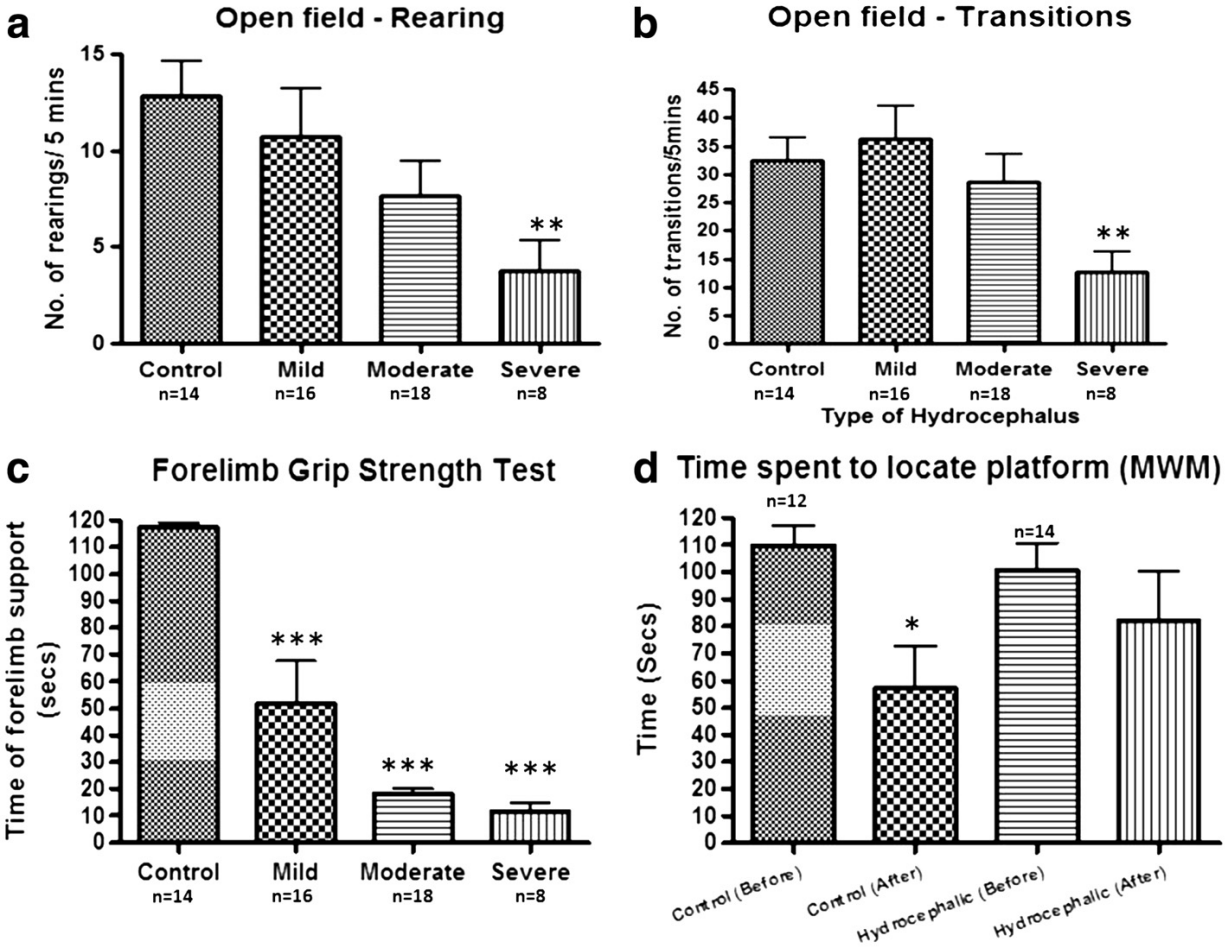
**Behavioural tests.** Histograms of behavioural tests in the control group and three groups of hydrocephalic rats. **a:** Vertical movements measured as number of open field rearings, **b:** horizontal movements measured as number of open field transitions, **c:** forelimb muscular strength measured in the forelimb grip strength test as the length of time the rats remain suspended and **d:** learning and memory in the Morris water maze (MWM). Movements and forelimb strength were gradually reduced as ventricular size increased. For the MWM, the time to reach the platform was tested before and after the rats had been through the training program. Learning was impaired in hydrocephalic rats. * p<0.05, ** p<0.01, *** p<0.001 vs control. Data are means ± SEM.

*Behavioural tests:* Open field test: The hydrocephalic rats were less active in horizontal exploratory as well as vertical movements (rearing) compared to controls. The reduction in rearing activities was more pronounced with increasing ventriculomegaly **,***p<*0.01 between control and severely hydrocephalic rats, but the overall correlation was not statistically significant (F = 2.723, R^2^-0.1335, *p* > 0.05, Figure 2a). The number of transitions or horizontal movements made was also reduced with increasing ventriculomegaly and was significant between the controls and the severely hydrocephalic rats (t = 3.145, *p<*0.01) (Figure 2b).

Forelimb grip strength test: This test which is a measure of the muscular strength in the forelimbs revealed a reduction of muscular strength, shown by a significantly shorter drop-off time in hydrocephalic rats compared with controls (*p<*0.001 for all groups). This also showed a graduated response between the various groups of hydrocephalus (F =52.83, R^2^-0.8066, *p<*0.001) (Figure 2c).

Modified Morris water maze test: After they had been through the training program, the control rats located the hidden platform in a shorter time than the hydrocephalic rats, suggesting impaired learning in hydrocephalic rats. There was a significant reduction in the escape latencies for the control rats compared to the hydrocephalic rats after training (t = 2.378 df = 8, *p<*0.05) (Figure 2d).

### Gross examination of the brain

**Figure 3:**
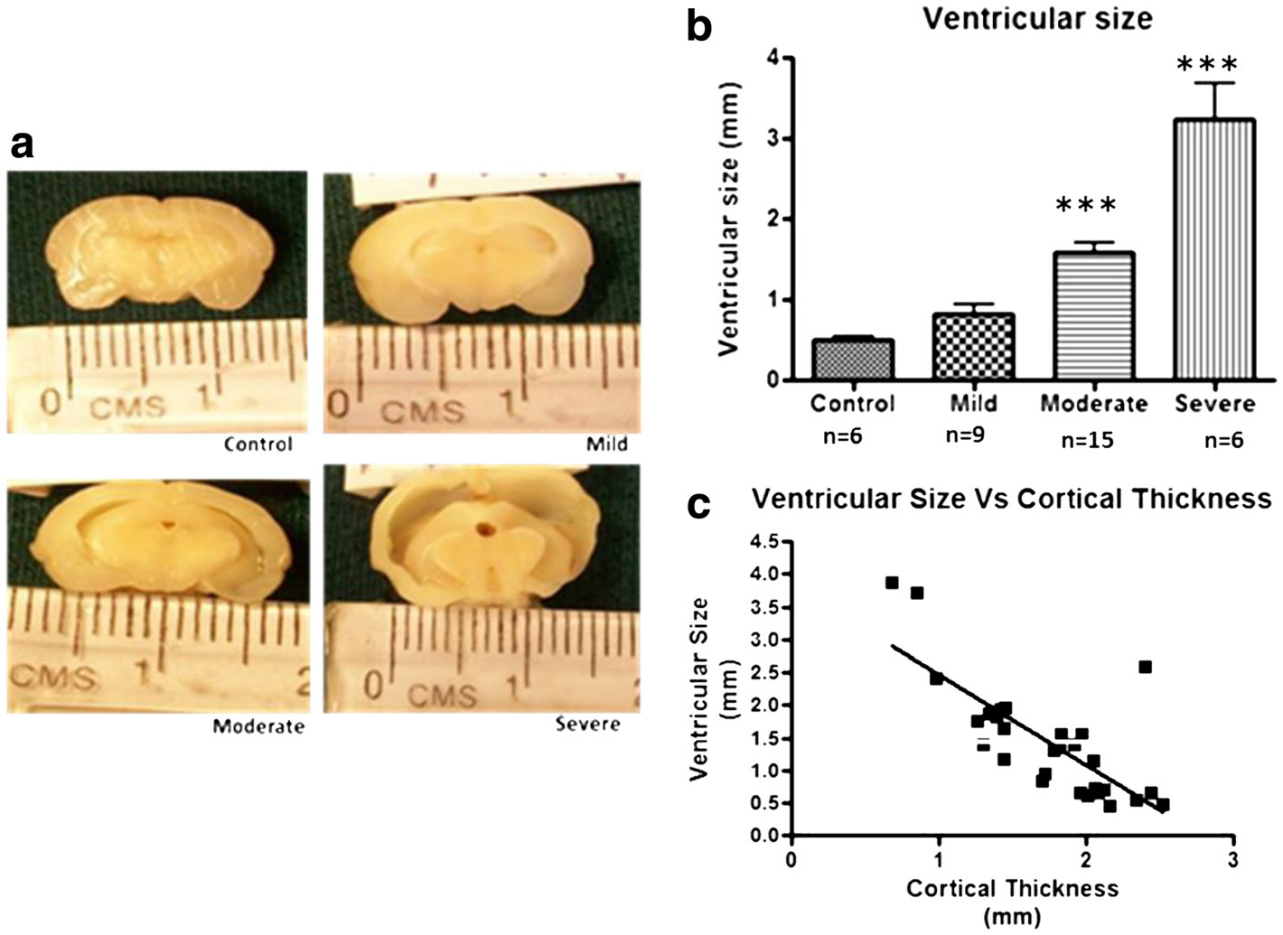
**Measurements of ventricular size in rats with mild, moderate and severe ventriculomegaly. a:** Photographs of fixed brain samples of controls and rats with different degrees of ventriculomegaly. **b:** Histogram of mean ventricular size in the different groups of hydrocephalic rats. (data are means ± SEM; *** *p<*0.001 vs control). **(c)** Graph showing the inverse correlation between the ventricular diameter and thickness of the cortical mantle (slope = −1.388 ± 0.2296, r^2^ = 0.5664, *p<*0.001). Ventriculomegaly is associated with progressive thinning of the cortical mantle.

In the experimental (kaolin-injected) rats, the degree of ventricular dilatation varied between animals. Ventriculomegaly was categorized as mild, moderate or severe according to the previously-mentioned criteria (Figure 3a). Ventricular size was significantly different for the four groups (F = 30.65, *p<*0.001). The moderate and severe groups were significantly larger than controls ( *p<*0.001 for both, Figure 3b). Ventricular size was inversely related to the thickness of the cortical mantle (Figure 3c).

### Histological examination

**Figure 4:**
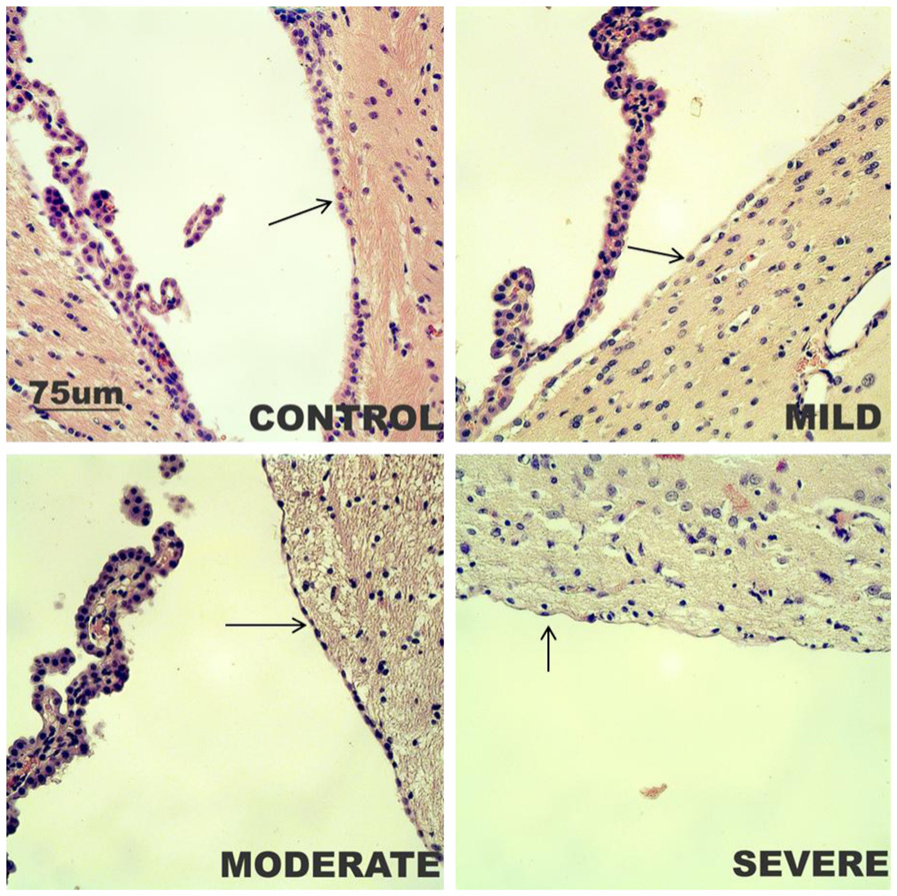
**Comparison of hematoxylin and eosin staining of brain samples.** Photomicrographs of the ependymal lining to the lateral ventricle in the control and hydrocephalic rats; note the progressive flattening of the ependymal cells with increasing ventriculomegaly (arrows). Scale bar (75 μm) is the same for all the photomicrographs.

**Figure 5:**
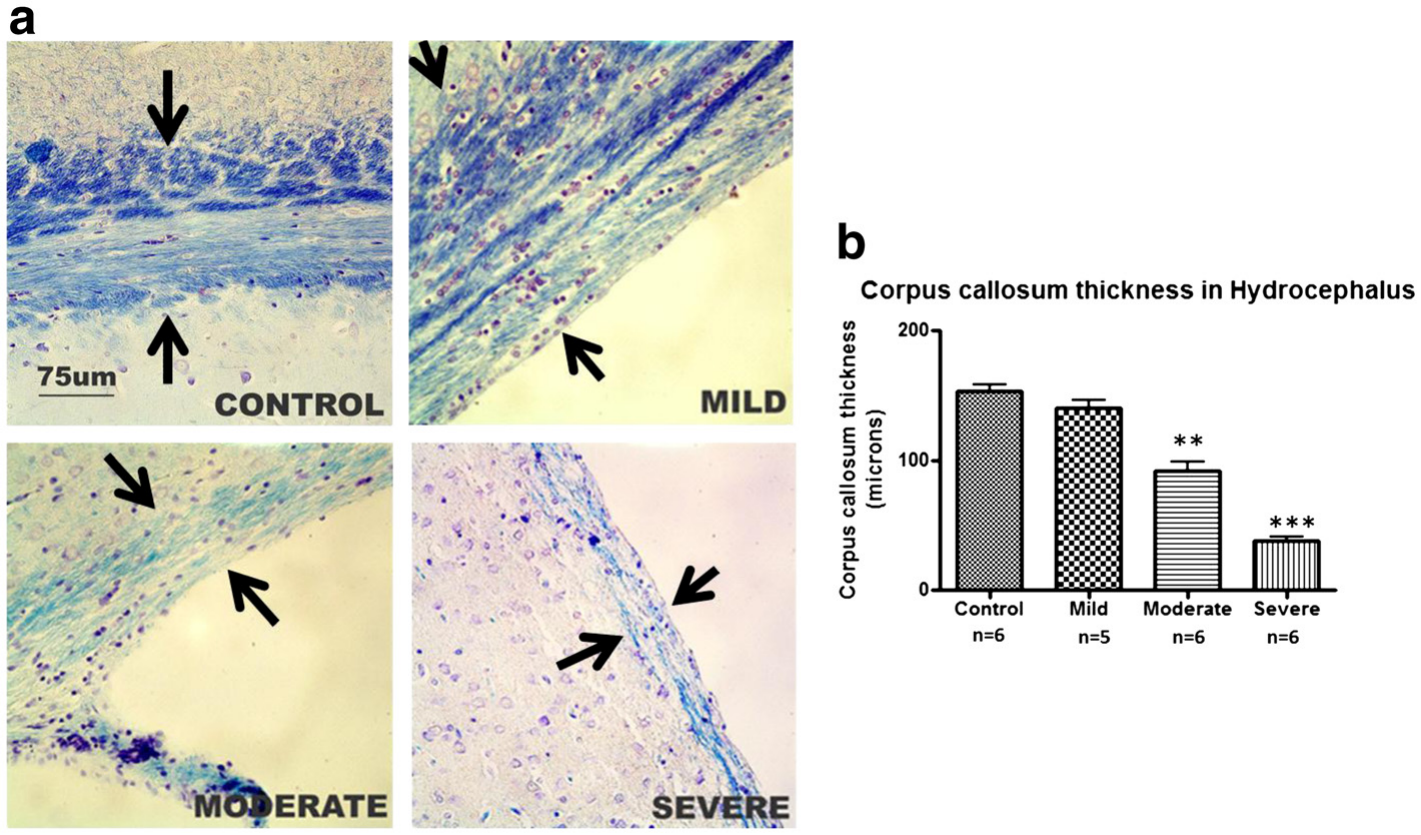
**Thickness of the corpus callosum in different grades of hydrocephalus. a:** Photomicrographs of the corpus callosum stained with luxol fast blue for myelin. Arrows indicate the site for thickness measurement **(b)** Histogram of the thickness of corpus callosum in the control and hydrocephalic brains (* *p<*0.05, ** *p<*0.001 vs control, data are means ± SEM). Ventriculomegaly is accompanied by gradual thinning of the corpus callosum. (Scale bar 75 μm).

Examination of the sections of the hydrocephalic brains revealed stretching/ flattening of the ependymal cells (Figure 4) with loss of cilia; cilia loss appeared to be more pronounced with increasing ventriculomegaly **(**not shown). There was also progressive thinning of the cerebral cortex with increasing ventricular dilatation. LFB staining of the myelin in the brain sections revealed progressive thinning of the corpus callosum and increasing extracellular space in the white matter with increasing ventriculomegaly (Figure 5a). The thickness of the corpus callosum as it courses round the lateral ventricle was significantly different between the various groups (F = 20.7, R^2^ = 0.795, *p<*0.001). The difference from control rats was significant in the moderate and severely hydrocephalic rats ( *p<*0.05and *p<*0.01, respectively, Figure 5b).

### Immunohistochemical examination

**Figure 6:**
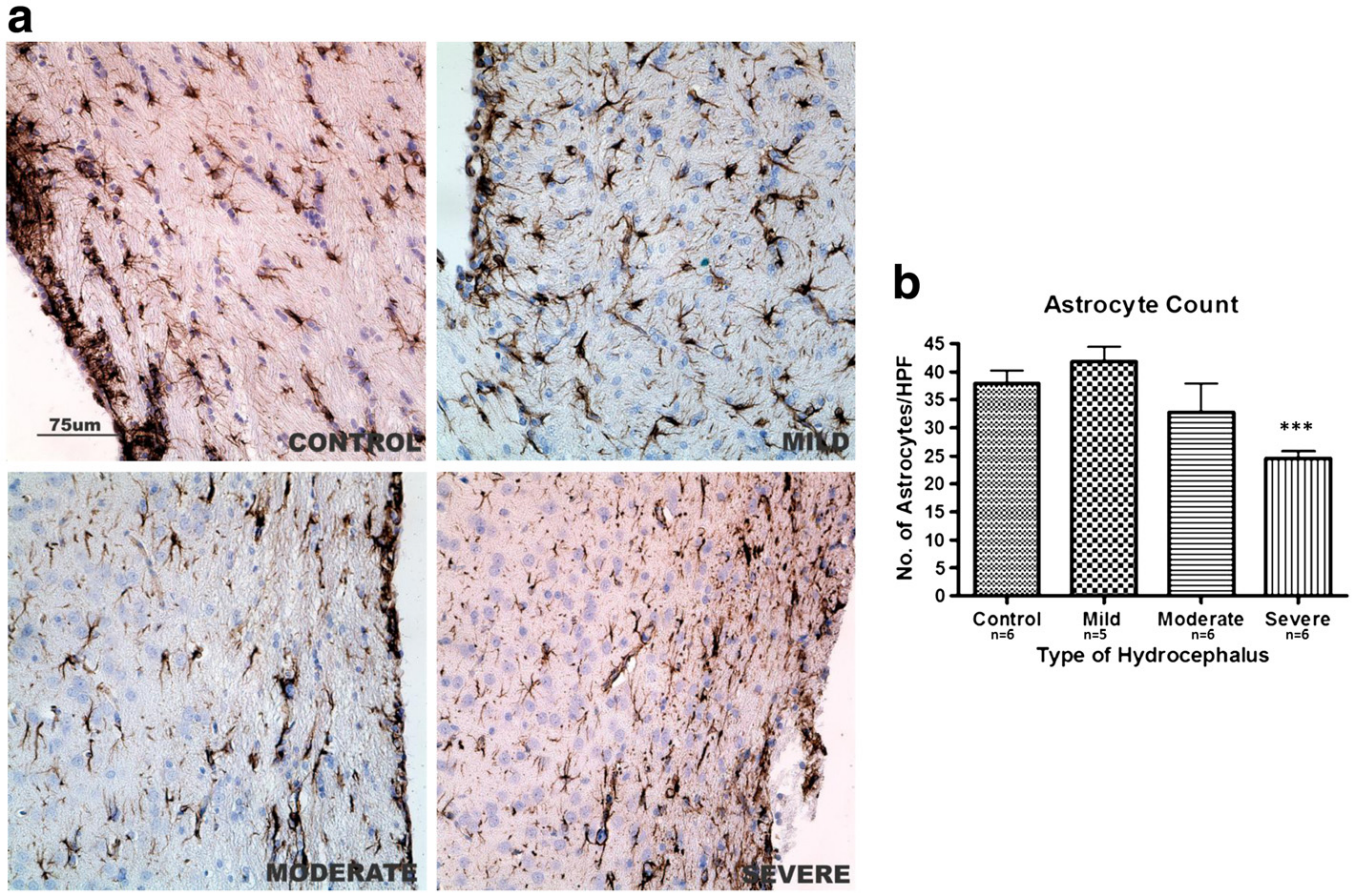
**Astrocytic activation in hydrocephalus. a:** GFAP immunoreactive cells in the sub-ependymal layer showing astrocytic activation (arrows) in the hydrocephalic rats **,** particularly in the mild group. **b:** Astrocyte count in the same region. Astrocyte count was raised in mild hydrocephalus (not significant) but gradually decreased as ventriculomegaly progressed and was significantly reduced in severe hydrocephalus (*** *p<*0.001 vs control). Data are means ± SEM. (HPF = High power field). Scale bar 75 μm).

In the control brain samples, the astrocytes were seen as scattered stellate cells in the subependymal layer and cerebral cortex. Reactive astrocytosis, seen as hypertrophy of the astrocytes, was observed in mildly hydrocephalic rats both in the subependymal layer and adjacent cortex; however, overall astrocytic count was reduced in the severe group compared to control (*p<*0.001, Figure 6a and b).

**Figure 7:**
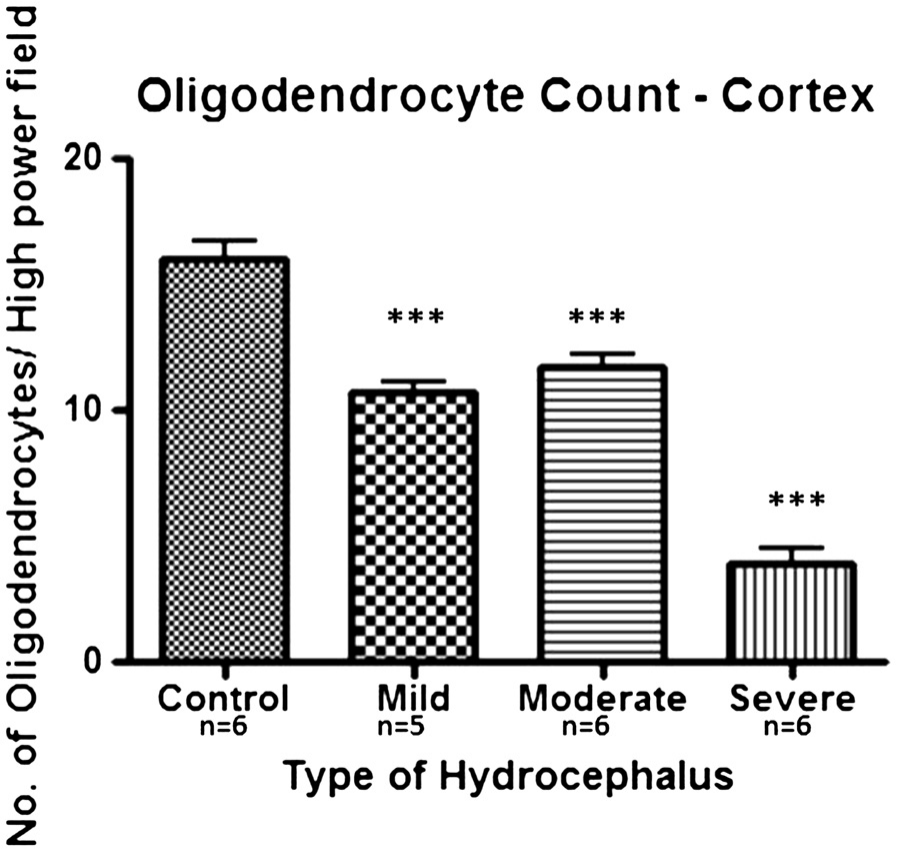
**Oligodendrocytes in hydrocephalus.** Oligodendrocyte count in the parietal cortex. Note marked reduction in number of CNPase immunoreactive cells especially in the severely hydrocephalic brain samples. ( ***p<0.001vs control).

Oligodendrocytes were labeled with anti-CNPase. CNPase is a myelin-associated enzyme expressed at high levels in myelin-producing cells: oligodendrocytes and Schwann cells. Oligodendrocytes were scattered in the cerebral cortex and more concentrated in the corpus callosum and sub-ependymal white matter in the control brain samples. There was a significant reduction in the population of oligodendrocytes both in the subependymal layer and the cerebral cortex of hydrocephalic brains corresponding with the severity of the hydrocephalus. All three hydrocephalic groups had significantly reduced numbers of stained cells in the cerebral cortex compared to controls (*p<*0.001) but this was most marked in the severely hydrocephalic rats (Figure 7).

**Figure 8:**
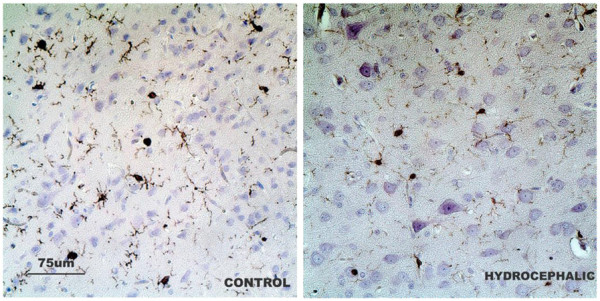
**Microglia activation in hydrocephalus.** Iba-1 immunoreactive cells (microglia) in the parietal cortex overlying the ventricles in the hydrocephalic rats and controls. Note withdrawal of dendritic branches in hydrocephalic rats. (Scale bar 75 μm).

Iba-1 staining of the control brain samples revealed scattered, non-activated microglia in the cerebral cortex seen as cells with extensive cytoplasmic branching. However, in the hydrocephalic rat brains, it revealed activated microglia seen as cells with very much reduced cytoplasmic branching (Figure 8). This response was observed in all the hydrocephalic brains but the overall microglial numbers were not altered between groups.

## Discussion

It has long been recognized that intracisternal injection of kaolin is an effective method for producing hydrocephalus in rodents and that the result is variable and somewhat unpredictable [13]. In this study, intracisternal injection of kaolin resulted in the development of hydrocephalus in about 90% of the rats. Despite the fact that the same volume of kaolin was injected, the rats developed varying degrees of ventricular dilatation which we have classified into mild, moderate and severe. The brains of the 3-week old rats used in this study were developmentally similar to that of a human infant [14]. Cisternal injection of kaolin causes a chemical arachnoiditis and results in obstructive hydrocephalus from impairment of flow of cerebrospinal fluid in the basal cisterns; this model is therefore similar to post-meningitic hydrocephalus in the human infant. With neonatal meningitis being one of the commonest causes of hydrocephalus in Africa [15], this is an appropriate experimental model for studying this disease. After an initial weight loss, hydrocephalic rats regained weight at the same velocity as controls, but mean body weight remained lower. This was most likely due to loss of appetite and reduced feeding activity in the early phase of induction of hydrocephalus. Del Bigio [16] has previously stated that delayed growth is one of the first signs of hydrocephalus onset.

The reduced motor activity of the hydrocephalic rats in the open field was most effectively illustrated by a significant reduction in vertical movements (rearing). Whereas the animals were freely mobile and exhibited preservation of horizontal movements, the reduced tendency for exploratory rearing may also be manifestation of hindlimb motor weakness**,** which could be due to damage of the periventricular axons. Although rearing activity was correlated with ventricular enlargement, the overall association was not statistically significant. This may be an area requiring further exploration, given the proximity of lower limb fibres to the ventricular system in mammals. The forelimb grip test, which directly measures the muscular strength in the forelimbs, lends weight to the observations in the open field test. The graduated reduction in muscular strength as the ventricular size increased observed here is similar to that reported from previous studies which used other tests of motor function like the rotating cylinder and ability to transverse a narrow beam [7,17]. Hydrocephalus is associated with damage to axons in the periventricular white matter including long tracts that project to the spinal cord [18], and manifests as a delayed myelination process in neurologically immature rats [2]. The relationship between ventricular distension and forelimb motor control has not been extensively studied. Our findings suggest that in this model, the forelimb grip test is a valuable measure of the impact of hydrocephalus on motor function in the proximal limbs. Learning and memory, as tested by the Morris water maze was observed to be impaired. The impairment of spatial learning ability [19], which was shown by the prolonged escape latency observed in the hydrocephalic rats, suggests that hydrocephalus impairs the function of the hippocampus and its connections i.e. fimbria, alveus and fornix.

The progressive flattening and discontinuation of the ependymal lining with increasing ventriculomegaly was distinct. From cuboid cells with cilia and round nuclei, the cells gradually lost their cilia and became flattened, stretched or disrupted with gaps seen in the epithelium in severe hydrocephalus – this was most obvious at the dorso-lateral angle of the lateral ventricle. The subependymal layer showed periventricular reactive astrogliosis with hypertrophy and a non-significant increase in the number astrocytes in the mildly hydrocephalic rats; however it was decreased in the moderate and severely hydrocephalic rats. This could mean that the neuroinflammatory process diminishes as the hydrocephalic process progresses; it could also be due to a generalized apoptosis taking place as hydrocephalus progresses, which affects all the brain cells including astrocytes. Fukushima *et al.*[20] reported an increase in production of new brain cells which were almost exclusively differentiated into astrocytes in hydrocephalic rats; however the study did not distinguish between the various degrees of ventriculomegaly. The progressive thinning of the corpus callosum with increasing ventriculomegaly implies that there is loss of the periventricular white matter secondary to pressure from the expanding ventricles. Oedema of the periventricular white matter with consequent enlargement of the extracellular spaces occurred in keeping with findings from other studies [18]; this is most likely due to stasis of extracellular fluid which normally drains into the CSF. These spaces increase as the ventriculomegaly progressed and thus demonstrates that the more severe the accumulation in the ventricles, the worse the oedema.

Oligodendrocytes in the periventricular white matter were reduced in quantity as the ventriculomegaly increased and the corpus callosum thinned out. The adjacent parietal cortex also showed this decline in oligodendrocytes which was most marked in the severely hydrocephalic rats. The loss of oligodendrocytes in hydrocephalus has been shown by Del Bigio and Zhang [21] to be due to cell death, caused by chronic ischaemia, toxicity from accumulation of glutamate, and inflammatory white matter injury.

Proliferation of astrocytes usually occurs secondary to central nervous system injury [22] and has been reported in hydrocephalus. In this study, the increased GFAP staining in the periventricular regions suggest that the periventricular white matter is an important site of brain injury in hydrocephalus, possibly from the stretch and compression which accompanies ventriculomegaly. Other factors such as hypoxia, ischaemia and cellular death, all present in hydrocephalus, could also be contributory. Gliosis occurred in both gray and white matter, but was more marked in the periventricular white matter. The role of periventricular astrocytic proliferative change in hydrocephalic brain injury is supported by studies that demonstrated increased brain GFAP ribonucleic acid (RNA) levels (measured by western blot) with progression of hydrocephalus in kaolin models of kittens [23], and increased CSF levels of GFAP in human hydrocephalic patients [24].

Reactive astrocytes secrete inhibitory molecules such as proteoglycans which create a hostile environment for remyelination of injured axons [25]. Together with microglia cells, the astrocytes also form a glial scar (that is, astrocytic proliferation and intracellular accumulation of glial filaments) which may play a major role in the chronicity of hydrocephalus in children as their brains are less pliable than normal [26]. There are similar reports of astrocytic activation observed in hydrocephalic Texas (H-Tx) rat models of congenital hydrocephalus [26]. Significantly increased expression of common pro-inflammatory cytokines have been reported in structures distant from the kaolin deposits but adjacent to the expanded ventricles such as the frontal cortex, parietal cortex, caudate- putamen, and hippocampus with normal expression in the medulla immediately adjacent to the kaolin deposits [27]. This lends weight to the belief that the neuroinflammation observed is secondary to the hydrocephalic process and not the direct consequence of kaolin injection. This has implications for putative pharmacological treatment of hydrocephalus.

Microglial cells are the primary immune effector cells of the brain parenchyma. They play a major role in the response to brain injury from a variety of causes including hydrocephalus. The characteristic morphological changes in microglia activation, that is, an altered morphology with withdrawal of the dendritic branches were observed in this study.

The brain parenchyma and periventricular white matter suffer various insults from the hydrocephalic process. The pressure from the fluid stretches the overlying cortex, stretches and sometimes damages the axons through ischaemia, the process of remyelination is limited by astrocytic proteoglycans and glial scar formation and the oligodendrocytes themselves are also reduced in number from necrosis/ apoptosis [16,21,28]. Each one of these processes needs to be addressed for the management of the hydrocephalic patient to be successful. Death of the oligodendrocytes is pivotal as they are needed for a successful remyelination process and since they do not seem to be regenerated [20,21], intervention needs to be timely.

In this study, the hippocampus appeared grossly normal and its cell population seemed unaltered on routine H&E staining (figure not shown), however, it was clearly functionally altered as manifested by the impairment in spatial learning and memory. This is however not surprising since changes may be substructural. It has been shown that biochemical composition and synaptic potentials are altered in hippocampal neurons in hydrocephalic rats [29]**.** Immunochemical staining of cholinergic neurons in the hippocampus, revealed a strong negative correlation between ventricular size, population of AChE (acetylcholinesterase) and ChAT (choline acetyltransferase) – immunosensitive cells in the hippocampus and spatial memory impairment [30]. Tashiro *et al*. [31] also reported progressive functional impairment of glutamic acid decarboxylase (GAD), parvalbumin (PV) and calbindin D28K neurons in the cerebrum and hippocampus observed by immunostaining while Nissl staining of the same tissues revealed almost no morphological changes.

The periventricular white matter seems to be the primary target in hydrocephalus with oedema, thinning and reduced cell populations observed which correspond to the degree of ventriculomegaly. However, the parietal cortex is also thinned and compressed by the increased ventricular size.

## Conclusion

In kaolin-induced hydrocephalus in three-week old rats, variable changes in the corpus callosum, ependymal and sub-ependymal layers and parietal cortex paralleled varying degrees of ventriculomegaly. Most of these changes could be correlated with the observed behavioural responses, thus confirming the results of previous studies reporting that the behavioural changes observed in hydrocephalus correspond with the degree of ventriculomegaly present.

## Competing interests

The authors declare that they have no competing interests.

## Authors’ contributions

FEO was involved in the design of the study, generation and analysis of data and drafting of the manuscript. MTS conceived and designed the study supervised the experiments and coordinated the preparation of the manuscript. A-LS was involved in the immunohistochemical staining and data analysis. All authors read and approved the final manuscript.
